# Intraclass Correlation and Variance in the Characteristics of Primary Care Patients Managing Chronic Medical and Behavioral Conditions

**DOI:** 10.7759/cureus.30970

**Published:** 2022-11-01

**Authors:** Abigail Crocker, Lisa W Natkin, Peter Callas, Levi Bonnell, Jessica Clifton, Juvena Hitt, Benjamin Littenberg

**Affiliations:** 1 Statistics, University of Vermont, Burlington, USA; 2 Human Services Research, University of Vermont, Burlington, USA; 3 Biostatistics, University of Vermont, Burlington, USA; 4 General Internal Medicine Research, University of Vermont, Burlington, USA

**Keywords:** variance, adult patients, statistical power, primary care, intracluster correlation

## Abstract

Background

To avoid statistical errors, researchers who recruit patients from selected medical practices and analyze them at the individual level need to account for the clustered nature of their sample. This is most often done using the intraclass correlation coefficients (ICCs), a measure of how strongly subjects recruited from the same cluster (in this case patients from a clinic) resemble each other.

Aims

The aim is to support the design of cluster-randomized studies by supplying estimates of variance and ICC of various measures using a population of patients from multiple primary care clinics.

Materials and methods

ICCs were extracted from a large cluster-randomized pragmatic clinical trial of adult primary care patients managing multiple chronic conditions, the Integrating Behavioral Health and Primary Care study (IBH-PC). IBH-PC collected demographics and patient-reported health outcomes on over 3,000 adults from 44 primary care practices in 13 states across the US. We present estimates of the standard deviation and ICC for gender, race, ethnicity, marital status, employment, income, education, social determinants of health, PROMIS-29 functional status, Duke Activity Status Index (DASI), nine-item Patient Health Questionnaire (PHQ-9) depression score, Generalized Anxiety Disorder (GAD-7) anxiety score, Asthma Symptom Utility Index, restricted activity days, medication adherence, health care visits in the past month, emergency room visits in the past year, hospital days in the past year, perception of quality and patient-centeredness of care, alcoholic drinks per month, and the GAIN substance use disorder screener.

Results

ICCs varied broadly with the highest values found for race and income and the lowest for short-term estimates of the GAIN.

Conclusions

These values can be used to inform the design, especially power estimates and sample size requirements, of future studies.

## Introduction

As more medical research studies use multi-level or clustered sampling designs, there is an increasing need for the use of intraclass correlation coefficients (ICCs) during sample size calculation [1]. ICC is a measure that “accounts for the relatedness of clustered data by comparing the variance within-cluster with the variance between clusters” [2]. Using an ICC to calculate sample size helps account for how strongly patients recruited from the same cluster (perhaps the same practice, school, class, family, or neighborhood) resemble each other, which effectively reduces the degrees of freedom. Not accounting for ICC during a sample size calculation will likely lead to errors, including an overestimate of effective sample size, falsely narrow confidence intervals around the measure of association, and errors in significance tests.

To avoid statistical errors, researchers who recruit patients from selected medical practices and analyze them at the individual level need to account for the clustered nature of their sample when they design multilevel or clustered studies [2]. Therefore, estimates of the ICC for variables of interest are necessary to perform an accurate sample size calculation. While ICCs have been published for primary care patients with certain conditions including diabetes [1,3] and heart disease [4], more information is needed on ICCs for outcome measures among patients with multiple chronic medical conditions [3,5,6]. Therefore, we sought to calculate ICCs for adults with multiple chronic conditions (MCC) in primary care.

## Materials and methods

This study was embedded in a large cluster-randomized pragmatic clinical trial of adult primary care patients managing MCC, the Integrating Behavioral Health and Primary Care study (IBH-PC) [7]. IBH-PC collected demographics and patient-reported health outcomes on over 3,000 adults from 44 primary care practices in 13 states across the US. The practices were a mix of Family Medicine, General Internal Medicine, and Community Health Center settings. Each had at least one licensed Behavioral Health Provider on site working for at least 50% effort at the time of data collection. As described elsewhere [7], we reviewed electronic health records to identify eligible subjects who were at least 18 years old, actively participating in primary care (at least two visits within 24 months with at least one within six months) and had an eligible medical and behavioral MCC or at least three MCC of either kind [7].

Data collection

Eligible patients were invited by mail with telephone follow-up to participate by mail. They could complete consent and data collection via a written postal survey, telephone interview, or a secure website. All materials and interviews were available in both English and Spanish. We present results from 2,953 eligible subjects who provided analyzable answers at the IBHPC baseline survey between September 2017 and May 2019.

Demographics and Social Characteristics

Age was recorded to the nearest year. Less than a quarter of 1% of respondents recorded a gender other than male or female. We collapsed self-reported race and ethnicity into two categories: Non-Hispanic white and all others. Marital status was collapsed into two categories: married or living as married vs. all others (never married, single, widowed, divorced, or separated). We reported employment status as employed (full-time, part-time, homemaker, student) vs. all others (retired, disabled, looking for work, and others). Income was recorded in seven ordered self-reported categories from less than US$15,000 per year to $100,000 per year or more and reported as a percent with income <$30,000 per year. Education was recorded as the highest level completed in seven categories from “Less than 9th Grade” to “Graduate or Professional Degree” and reported as the percent with any college education. Self-reported social determinants of health were recorded as insecurities over the past 12 months in three categories: food, housing and living expenses.

Functional Status

The Patient-Reported Outcomes Measurement Information System (PROMIS -29) [8] is a validated self-report health measure. The PROMIS-29 consists of two global scores (Physical and Mental) as well as eight domain scores (Fatigue, Pain Interference, Pain Intensity, Sleep Disturbance, Social Participation, Anxiety, and Depression). Seven of the domain scores consist of four items each on a five-point Likert scale (very poor to very good). Pain Intensity is based on a single item with a numeric range from 0 to 10. Higher items indicate more of each of the subscales and the global scores.

The Duke Activity Status Index (DASI) [9] is a 12-item validated self-report measurement used to assess functional capacity. Questions include an assessment of engagement in daily household chores (e.g., dusting, raking leaves) and of various intensities of physical exercise (e.g., running, golfing, swimming). Answers choices are dichotomized as “yes” or “no.” Scores are weighted and can range from 0 to 58.2 with higher scores indicating greater levels of fitness.

The nine-item Patient Health Questionnaire (PHQ-9) is a validated self-report measure of depression symptom severity. [10] Questions include having “little interest or pleasure in doing things” and “feeling tired or having little energy” and are rated on a four-point Likert scale (not at all to nearly every day) over the past two weeks. Higher scores indicate more severe depression symptomology. A total score is calculated by summing up all items, scores range from 0 to 27.

The seven-item Generalized Anxiety Disorder Scale (GAD-7) [11] is a self-report questionnaire developed and validated in a larger primary care patient sample to assess anxiety symptom severity over the prior two weeks. Items include “feeling nervous” and “trouble relaxing” which are rated on a four-point Likert scale (not at all to nearly every day). Higher scores indicated more severe anxiety. A total score is calculated by summing up all items; scores range from 0 to 21.

The Asthma Symptom Utility Index (ASUI) [12] is a 10-item validated self-report measure assessing control and quality of life as it relates to asthma symptoms. Specifically, questions include an evaluation over the past two weeks regarding the frequency and severity of breathing difficulty (shortness of breath, chest tightness) and challenges with asthma medication. Frequency questions were rated on a four-point Likert scale (not at all, 1-3 days, 4-7 days, 8-14 days) and severity was rated on a three-point Likert scale (mild, moderate, severe). The ASUI is a weighted scale with a summary score from 0 to 1. Lower scores indicate more troublesome asthma symptoms. The ASUI was asked only of those subjects with asthma noted in their health record.

Patients were asked to report on the restriction of their daily lives due to illness and disability. Specifically, the question stated: “During the past 2 weeks, how many days did you miss at least a 1/2 day from a job, business, school or regular activity because of illness or injury.” [13]

Utilization, Perception of Care, and Adherence

Medication adherence was measured using the Morisky Green Levine Scale, a four-item self-report measure assessing adherence to prescription medication [14]. Questions include the following: “Do you ever forget to take your medicines?” and “Sometimes if you feel worse when you start taking the medicine, do you stop taking it?” Answer choices are dichotomized as yes or no. Summary scores indicate either low adherence (at least one “yes” response) or high adherence. The scale has been shown to have adequate reliability (Cronbach alpha = 0.6 [15]).

Healthcare utilization was estimated by asking patients to recall the number of visits to the emergency room, days spent overnight in the hospital, and appointments with a healthcare provider. Response options were on a numeric scale ranging from 0 to 10, and “more than 10.”

The Patient-Centered Index is a 16-item questionnaire developed as part of the IBH-PC with the collaboration of researchers, patients, and other stakeholders to assess how patient-centered a patient perceives their care [16]. Questions are oriented around a patient’s practice and include, “they talk to me with respect,” “we discuss treatment options together,” and “I feel welcome.” Each item is scored on a five-point Likert scale from 0 to 4. The item scores are summed, multiplied by 25, and divided by the number of valid answers to yield a total score from 0 (low perceived patient-centeredness) to 100 (high perceived patient-centeredness).

The Consultation and Relational Empathy (CARE) measure is a 10-item validated self-report assessment of the quality of medical care [17]. Example questions include: How good is your practitioner at “making you feel at ease” and “really listening.” Items are rated on a five-point Likert scale (poor to excellent). A total score is calculated by summing all items. Higher scores indicate a higher quality of patient-perceived medical care.

Substance Use/Problem Drinking

Alcohol use was assessed using two items from the Self-Report Habit Index-Alcohol (SRHI-A) [18]. Specifically, these questions included, “during the past 30 days, how many days did you drink one or more alcoholic beverages” (response options: 0-30) and “on the days that you drank during the past 30 days, how many drinks did you usually have each day” (response options: <1, 1, 2-5, 6-15, 16-25, 26-35, >35). Three scores were calculated, including the frequency of drinking, the total number of drinks consumed in the past month, and high-risk drinking (i.e., ever having four or more drinks for women; five or more for men).

The Global Assessment of Individual Needs- Short Screener (GAIN-SS) [19]) is a biopsychosocial screener for individuals presenting with substance use and mental health concerns. We report on the 5-item Substance Disorders subscale. Responses are on a five-point Likert scale. Domain scores are calculated as the sum of the number of items endorsed by the respondent over each of four timeframes (past one month, past three months, past year, lifetime). Higher scores indicate more severe symptoms. The GAIN-SS was asked only of those subjects with substance use disorder noted in their health record.

Statistical analysis

Within the framework of analysis of variance (ANOVA) and random effects models, the ICC (sometimes labeled with the Greek letter rho, ρ) is the proportion of the overall variance explained by the between cluster (practice) variance. The ICC will be close to zero if patients within a practice are no more similar with regard to an outcome measure than patients at a different practice. Alternatively, if patients within a practice are very similar in terms of an outcome measure, the ICC will be close to one and the effective sample size will be close to the number of practices. In general,

$$ICC = \sigma _{b}^{2}/(\sigma _b^2+\sigma_w^2)$$

where b is the between-cluster component of variance and w is the within-cluster component.

We used the Stata loneway command which uses the F statistic to calculate ICC for the total sample size N, mean sample size per cluster $N_0$, and k practices. 

$$ICC = \frac{F_{obs}-1}{F_{obs}-1+g}$$

where

$$g = \frac{N-\sum_i\frac{N_o^2}{N}}{k-1}$$

The asymptomatic standard error of the ICC is

$$\sqrt{(V)(ICC)}$$

and the 100(1-α)% confidence interval is

$$ICC \pm z_{\sigma/2}\sqrt{(V)(ICC)}$$

The total sample size (N), mean sample size per cluster ($N_0$), mean (for continuous variables), proportion (for dichotomous variables), range (for non-binary categorical variables), standard deviation (for continuous variables), standard error of the mean accounting for clustering, and the ICC with 95% confidence intervals were reported for each outcome measure in the study.

The protocol was approved by the University of Vermont Institutional Review Board’s Committee on Human Research (CHRMS#16-554). Analyses were performed in Stata 16.1 (Stata Corp., College Station, TX).

## Results

Social and demographic variables such as race (0.25) and income (0.21) had the highest ICC values, reflecting the relatively homogenous nature of each practice’s catchment area. The smallest degree of clustering was observed for the short-term Global Assessment of Individual Needs, especially for the shorter-term subscales, which showed essentially no within-practice correlation. The other characteristics analyzed had values between these extremes (Table 1).

**Table 1:** Variance and Intracluster correlation coefficients N = sample size; m = mean sample size per cluster; sd = standard deviation; se = standard error; ICC = Intracluster correlation coefficient; CI = confidence interval; PROMIS = Patient-Reported Outcomes Measurement Information System; PHQ = Patient Health Questionnaire; GAD = Generalized Anxiety Disorder Scale; CARE = Consultation and Relational Empathy (CARE) measure; GAIN = Global Assessment of Individual Needs.

Domain	N	m	mean	sd	se	ICC	95%	CI
Demographic and social characteristics								
Age	2,945	71.8	61.8	13.3	0.2	0.080	0.040,	0.120
Male gender	2,953	68.7	35.8%	--	0.9%	0.006	0.000,	0.014
White race	2,901	69.1	76.7%	--	0.8%	0.254	0.161,	0.348
Hispanic ethnicity	2,913	69.4	8.3%	--	0.5%	0.123	0.067,	0.179
Married or living as married	2,936	71.6	46.4%	--	0.9%	0.087	0.044,	0.130
Employed, homemaker or student	2,835	69.1	32.8%	--	0.9%	0.017	0.003,	0.031
Income	2,796	68.2	53.5%	--	0.9%	0.207	0.125,	0.289
College participation	2,874	70.1	45.3%	--	0.9%	0.114	0.062,	0.167
Food Insecurity	2,874	70.1	12.7%	--	0.6%	0.028	0.009,	0.047
Housing Insecurity	2,881	70.3	3.4%	--	0.3%	0.007	0.000,	0.016
Financial Insecurity	2,855	69.6	24.4%	--	0.8%	0.046	0.020,	0.073
Functional status								
PROMIS-29 Social Participation	2,945	71.8	48.1	10.0	0.2	0.046	0.020,	0.072
PROMIS-29 Anxiety	2,945	71.8	54.1	10.1	0.2	0.059	0.027,	0.090
PROMIS-29 Depression	2,945	71.8	53.0	9.8	0.2	0.057	0.026,	0.088
PROMIS-29 Fatigue	2,945	71.8	52.7	10.4	0.2	0.027	0.009,	0.045
PROMIS-29 Pain Interference	2,945	71.8	58.3	10.1	0.2	0.059	0.027,	0.091
PROMIS-29 Physical Function	2,945	71.8	43.2	9.5	0.2	0.070	0.034,	0.107
PROMIS-29 Sleep Disturbance	2,945	71.8	53.2	8.9	0.2	0.037	0.015,	0.059
PROMIS-29 Pain Intensity	2,945	71.8	4.5	2.8	0.1	0.105	0.056,	0.155
PROMIS-29 Physical Health Summary	2,945	71.8	45.5	9.5	0.2	0.073	0.036,	0.110
PROMIS-29 Mental Health Summary	2,945	71.8	50.1	8.9	0.2	0.060	0.028,	0.092
Duke Activity Status Index (DASI)	2,727	66.5	29.5	16.1	0.3	0.060	0.028,	0.092
PHQ-9 total score	2,859	69.7	6.6	6.1	0.1	0.058	0.027,	0.090
PHQ-9>4	2,859	69.7	51.0%	--	0.9%	0.049	0.021,	0.076
GAD-7 total score	2,887	70.4	4.6	5.3	0.1	0.064	0.031,	0.098
GAD-7>4	2,887	70.4	37.9%	--	0.9%	0.045	0.019,	0.071
Asthma Symptom Utility Index	556	13.6	0.8	0.2	0.0	0.016	0.000,	0.057
Restricted activity days	2,867	69.9	1.2	2.5	0.0	0.025	0.007,	0.042
Utilization, perception of care and adherence								
Morisky Green Levine Adherence score	2,903	70.8	1.0	1.2	0.0	0.035	0.013,	0.056
Emergency room visits in past year	2,920	71.2	1.1	1.9	0.0	0.045	0.019,	0.071
Health care visits in past month	2,916	71.1	2.5	2.7	0.1	0.020	0.005,	0.034
Hospital days in past year	2,913	71.0	1.1	2.4	0.0	0.015	0.002,	0.027
Patient Centeredness Index	2,773	67.6	84.0	16.1	0.3	0.020	0.004,	0.035
CARE total score	2,925	71.3	4.3	0.9	0.0	0.027	0.009,	0.046
Substance use/Problem Drinking								
Drinks per month	2,142	52.2	12.6	58.5	1.3	0.005	0.000,	0.016
GAIN lifetime SUD screener	480	12.3	1.8	1.9	0.1	0.071	0.001,	0.141
GAIN 1-year SUD screener	480	12.3	0.7	1.2	0.1	0.007	0.000,	0.049
GAIN 3-month SUD screener	480	12.3	0.5	1.1	0.0	0.000	0.000,	0.039
GAIN 1-month SUD screener	480	12.3	0.5	1.0	0.0	0.000	0.000,	0.039

## Discussion

We present estimates of the standard deviation and ICC for 39 characteristics of adults with MCC seen in primary care settings. They can be used to inform the design, especially power estimates and sample size requirements, of future studies. The ICC may be used to calculate the design effect, DE, and how much the sample size must be inflated above that of a simple random sample to account for the loss of information inherent in the clustered design. If the mean number of subjects sampled per cluster is m,

$$DE = 1 +(M-1)\cdot ICC$$

If either m or ICC is large, the total number of individual subjects needed may be notably greater than suggested by a sample size calculation that is not adjusted for clustering. If both m and ICC are small, the design effect may be very close to 1.0, indicating that the clustered design does not inflate the sample size. Because design effects are dependent on the number of subjects per cluster, they do not translate easily to other settings. Needless to say, if the study design includes clustering at some level, it is important to consider adjusting for ICC in the sample size calculation. Therefore, we present ICCs to allow the ready calculation of the design effect.

Other catalogs of ICCs have been published [1,3,5,6,20-23] They demonstrate some variability in the estimates for variance and ICC that may be due to differences in the populations and settings studies. Therefore, investigators should be wary of using the smallest ICC available in estimating sample size requirements. For instance, the values presented here were drawn from a very broad population of adults in 13 states around the US, but excluded healthy adults, children, Veterans Affairs clinics, and practices without on-site behavioral health services.

The within-practice ICCs varied dramatically by domain with the largest values emerging from demographic and social characteristics. The White race had the highest value (0.254) followed by low income (0.207), Hispanic ethnicity (0.123), and college participation (0.114). We purposely selected different types of practices to be included in the study (large academic centers, private, FCQAs, community health centers) from different locations (urban, suburban, rural) and varying levels of wealth. Therefore, we would expect the patient populations to be different too. Perhaps the high demographic and social correlations may be representative of the populations that practices serve. Pain intensity (0.105) also had an ICC > 0.10. It is conceivable that some physicians are so risk-averse in prescribing opioid medications for chronic pain that they try to avoid these patients altogether. In contrast, correlations were lowest among the GAIN short-term SUD screener questions.

The generalization of these results may be the largest limitation of this study. Although the participants represented the US population in several key characteristics, the results may not be generalizable to other populations, such as those without MCC. Further, these results may not generalize to studies that clustered on factors outside of primary care clinics with co-located behavioral health services. However, different practice sizes and types were included (academic, private, non-profit) from geographically and socially diverse areas. Results may not generalize outside the 13 states represented in this study; however, all the major US regions were included. Future reports of ICCs should fully describe the data sources employed and populations studied to allow readers to judge the applicability of their findings to the readers' design tasks.

## Conclusions

The variance structures of clustered clinical trials, in which treatment is assigned to groups of subjects (clusters) rather than to individual subjects, are inherently more complex, often resulting in reduced statistical power. Methods to accommodate this in the analysis phase are well-developed. However, planning clustered trials can be difficult without some estimate of the impact of clustering on sample size requirements. If the subjects of interest are adults, especially those with MCC, the results published here may be helpful.
